# Beyond the expected: the cerebellum as a multifaceted player in mental disorders

**DOI:** 10.1055/s-0046-1816042

**Published:** 2026-02-27

**Authors:** Christian Messina

**Affiliations:** 1 Azienda Sanitaria Provinciale Catania, Catania, Italy.

Dear Editor,

We read with great interest the comprehensive review by Braga-Neto et al., which provides an in-depth overview of the association between structural and functional abnormalities of the cerebellum and a wide range of psychiatric disorders, including schizophrenia, bipolar disorder, depression, anxiety disorders, attention-deficit/hyperactivity disorder (ADHD), and autism spectrum disorder (ASD). 1 Their work, supported by clinical evidence as well as functional and structural imaging studies, highlights the cerebellum's involvement across cognitive, emotional, and behavioral domains. 1 We would like to congratulate the authors for this thorough contribution and, at the same time, add our perspective to further enrich the discussion on the role of the cerebellum within the spectrum of psychiatric disorders.

In addition to the psychiatric conditions discussed in the review, prior studies have also highlighted the involvement of the cerebellum in obsessive-compulsive disorder (OCD). While early models of OCD pathophysiology were largely centered on the cortico-striato-thalamo-cortical (CSTC) circuit, neuroimaging research over the past two decades has increasingly demonstrated consistent cerebellar abnormalities associated with this disorder. 2

Importantly, these alterations do not appear in isolation but rather interact with basal ganglia structures and CSTC pathways, thereby expanding and refining the traditional model. 2 In particular, disrupted connectivity has been reported between the cerebellum and the caudate/putamen, as well as between the cerebellum and dorsolateral or ventrolateral prefrontal cortices. Both the right and left cerebellar hemispheres, and in some cases the vermis, have been implicated. 2 Collectively, these findings support the notion that the cerebellum represents a critical hub within the broader neural network underlying OCD symptomatology.

Furthermore, evidence has also pointed to cerebellar involvement in personality disorders, particularly in borderline personality disorder (BPD), which is characterized by profound instability in emotional regulation, interpersonal functioning, identity, and impulse control. 3 These domains are strongly linked to the prefrontal cortex (PFC), which maintains extensive reciprocal connections with the cerebellum. 3

Consequently, alterations in the cerebellar-PFC circuitry may significantly influence the clinical manifestations of BPD. 3 The cerebellum plays a key role in contextualizing both internal and external stimuli, integrating their temporal and spatial dynamics, and supporting the generation of coherent behavioral responses. 3 Disruption of these circuits, and of the feedback projections toward frontal and parietal regions, may hinder the ability to construct organized and context-sensitive behaviors. 3 Additionally, the cerebellum contributes to comparing cortical predictions with incoming sensory inputs, a mechanism essential for novelty detection and attentional shifting. 3 Impairment of these cortico-cerebellar loops could, therefore, explain the deficits in sensory prediction and novelty processing that have been described in individuals with BPD. 3

Additionally, emerging evidence indicates that the cerebellum is also implicated in the pathophysiology of posttraumatic stress disorder (PTSD). Structural studies have reported reduced cerebellar volumes and altered cerebellocortical connectivity in individuals with PTSD. 4 Functionally, decreased activation of the right lobule V has been observed during subliminal threat presentation, a finding that may contribute to emotional dysregulation in these patients. 4

Moreover, resting-state analyses revealed reduced functional connectivity of the left flocculus with cortical areas involved in bodily self-consciousness, including the temporo-parietal junction, supramarginal and angular gyri, and the superior parietal lobule. 4 Decreased connectivity was also detected between the left flocculus and medial prefrontal cortex, precuneus, and mid/posterior cingulate cortex—regions belonging to the default mode network. 4 Conversely, an increased functional coupling of the right flocculus with the right anterior hippocampus has been reported, a structure frequently impacted by early-life trauma and stress-related disorders. 4

The involvement of the cerebellum in eating disorders has also gained increasing attention. A reduced volume of the right crus I has been reported in patients with anorexia nervosa (AN), a finding thought to reflect impaired integration of cognitive, emotional, and behavioral inputs. 5 This deficit may limit the ability to incorporate new therapeutic strategies, thereby reinforcing rigid and inflexible patterns of behavior. 5 Such an interpretation is supported by animal studies, where disruption of right crus I function led to repetitive and perseverative behaviors. 5

Notably, reduced cerebellar volume has been associated with the presence of AN, increased vulnerability to its development, and with poorer treatment outcomes. 5 Moreover, smaller volumes have been correlated with higher levels of alexithymia in affected individuals. 5

In addition to structural alterations, intrinsic cerebellar network connectivity appears to be disrupted in patients with eating disorders when compared to healthy controls. 5 Specifically, increased connectivity with the insula, vermis, and paravermis, alongside reduced connectivity with parietal regions, has been observed. 5 Furthermore, disorder-specific patterns have emerged: patients with AN show stronger cerebellar-insular connectivity, while those with bulimia nervosa (BN) display enhanced connectivity with the anterior cingulate cortex. 5 These findings underscore the cerebellum's contribution to the complex cognitive-emotional mechanisms underlying eating disorders.

Taken together, the evidence from OCD, BPD, PTSD, and eating disorders converges on the view that the cerebellum acts as a cross-domain hub within the psychopathological spectrum. Current literature supports a nuanced, bidirectional interpretation.

On one hand, classical lesion studies and the well-characterized cerebellar cognitive-affective syndrome demonstrate that primary damage can produce disturbances of affect, executive function, and social cognition. This indicates that cerebellar dysfunction is capable of causally contributing to psychiatric-like symptoms. Indeed, lesion and stimulation studies indicate that isolated cerebellar pathology can generate cognitive and affective changes, supporting a causal role in certain contexts.

On the other hand, abundant evidence shows that neurotransmitter disturbances central to psychiatric disorders (for example, abnormalities of dopaminergic, glutamatergic and GABAergic signaling) directly affect cerebellar circuits and synaptic physiology. Thus, in many cases cerebellar alterations may arise downstream of widespread neurochemical imbalances.

Therefore, the most consistent model emerging from the literature is one of interaction: cerebellar abnormalities can be both a consequence of primary neurotransmitter or cortical-subcortical dysregulation and an independent contributor that, via altered cerebello-cerebral loops, amplifies or shapes clinical symptomatology. 2 This integrative perspective has practical implications, suggesting that interventions targeting cerebellar function (e.g., noninvasive brain stimulation) may modulate symptoms directly and could complement strategies aimed at restoring neurochemical balance. 3 These structural and functional alterations appear to modulate higher-order processes—including emotional regulation, cognitive flexibility, self-referential processing, and interoceptive awareness—suggesting that cerebellar dysfunction may represent a shared substrate across distinct psychiatric conditions.

## References

[1] Braga-Neto P Santos MWBd Scott S SO The cerebellum and psychiatric disorders: unraveling its role in mental health Arq Neuropsiquiatr 2025 83 08 1 8 10.1055/s-0045-1810408 PMC12399322 40886707

[2] Yang Y Xia Y Zhou Z Guo Z Tian L Cerebellum and obsessive-compulsive disorder: A narrative review of neuroimaging evidence from MRI studies Asian J Psychiatr 2025 110 104606 10.1016/j.ajp.2025.104606 40617144

[3] De Vidovich G Z Muffatti R Monaco J Repetitive TMS on Left Cerebellum Affects Impulsivity in Borderline Personality Disorder: A Pilot Study Front Hum Neurosci 2016 10 582 10.3389/fnhum.2016.00582 27994543 PMC5136542

[4] Rabellino D Thome J Densmore M Théberge J McKinnon M C Lanius R A The Vestibulocerebellum and the Shattered Self: a Resting-State Functional Connectivity Study in Posttraumatic Stress Disorder and Its Dissociative Subtype Cerebellum 2023 22 06 1083 1097 10.1007/s12311-022-01467-4 36121553 PMC10657293

[5] Milos G Kaufmann L K Jäncke L Does local cerebellar volume predict treatment success in anorexia nervosa? Psychiatry Res Neuroimaging 2021 317 111355 10.1016/j.pscychresns.2021.111355 34450453

